# Supramolecular Zn(II)-Dipicolylamine-Azobenzene-Aminocyclodextrin-ATP Complex: Design and ATP Recognition in Water

**DOI:** 10.3390/ijms22094683

**Published:** 2021-04-28

**Authors:** Shohei Minagawa, Shoji Fujiwara, Takeshi Hashimoto, Takashi Hayashita

**Affiliations:** 1 Department of Materials and Life Sciences, Faculty of Science and Technology, Sophia University, 7-1 Kioi-cho, Chiyoda-ku, Tokyo 102-8554, Japan; shohei-minagawa@eagle.sophia.ac.jp (S.M.); s-fujiwara@kanagawa-u.ac.jp (S.F.); 2 Department of Material and Life Chemistry, Faculty of Engineering, Kanagawa University, 3-27-1 Rokkakubashi, Kanagawa-ku, Yokohama-shi, Kanagawa 221-8686, Japan

**Keywords:** supramolecular complex, ATP recognition, cyclodextrin, dipicolylamine, circular dichroism

## Abstract

Cyclodextrins (CyDs) are water-soluble host molecules possessing a nanosized hydrophobic cavity. In the realm of molecular recognition, this cavity is used not only as a recognition site but also as a reaction medium, where a hydrophobic sensor recognizes a guest molecule. Based on the latter concept, we have designed a novel supramolecular sensing system composed of Zn(II)-dipicolylamine metal complex-based azobenzene (**1-Zn**) and 3A-amino-3A-deoxy-(2A*S*,3A*S*)-*γ*-cyclodextrin (3-NH_2_-*γ*-CyD) for sensing adenosine-5′-triphosphate (ATP). **1-Zn** showed redshifts in the UV-Vis spectra and induced circular dichroism (ICD) only when both ATP and 3-NH_2_-*γ*-CyD were present. Calculations of equilibrium constants indicated that the amino group of 3-NH_2_-*γ*-CyD was involved in the formation of supramolecular **1-Zn**/3-NH_2_-*γ*-CyD/ATP. The Job plot of the ICD spectral response revealed that the stoichiometry of **1-Zn**/3-NH_2_-*γ*-CyD/ATP was 2:1:1. The pH effect was examined and **1-Zn**/3-NH_2_-*γ*-CyD/ATP was most stable in the neutral condition. The NOESY spectrum suggested the localization of **1-Zn** in the 3-NH_2_-*γ*-CyD cavity. Based on the obtained results, the metal coordination interaction of **1-Zn** and the electrostatic interaction of 3-NH_2_-*γ*-CyD were found to take place for ATP recognition. The “reaction medium approach” enabled us to develop a supramolecular sensing system that undergoes multi-point interactions in water. This study is the first step in the design of a selective sensing system based on a good understanding of supramolecular structures.

## 1. Introduction

Supramolecular chemistry is defined as an assembly of molecules bound by multiple weak noncovalent forces to form supramolecular structures that are expected to exhibit novel functions not found in simple molecules [1,2]. In the realm of molecular recognition, conventional chemical sensors have adopted the concept of static molecular recognition, which is characterized by a one-to-one complexation between host and guest molecules, like a “lock-and-key model” [3]. If the concept of dynamic molecular recognition, e.g., the existence of several equilibria among host and guest molecules, is adopted in the design of a supramolecular sensing system, a unique recognition function not found in a simple static molecular recognition system is expected to be achieved.

Cyclodextrin (CyD), a macrocyclic oligosaccharide composed of _D_-glucopyranose units, is a water-soluble host molecule with a nanosized hydrophobic cavity. This characteristic has been used in various molecular recognition systems. Conventional CyD-based sensors are static molecular recognition systems that use the hydrophobic cavity as a recognition site. For example, displacement assays that utilize the difference in inclusion ability for a chromophore and a target molecule in CyD cavities have been reported [4,5,6,7]. CyDs are also used as a catalyst in the realm of organic chemistry, giving a reaction medium to a reactant [8,9]. Therefore, we speculated that the CyD cavity would be used as a “reaction medium” where molecular recognition takes place. The most important benefit is that a hydrophobic sensor can work in an aqueous medium. Our group has reported several examples of selective supramolecular sensing systems for sugars [10,11,12,13].

Phosphate derivatives, such as nucleoside phosphate and inorganic phosphate, play an important role in biological systems. Adenosine-5′-triphosphate (ATP) is a universal energy source for various cellular functions in living systems. In this regard, ATP sensor development is attracting much attention. CyD-based phosphate derivative sensors have been used in UV-Vis measurements [14,15] and electrochemistry [16], with quantum dots [17] and aptamers [18], and in fluorescence [19] and aggregation [20] studies. In addition, we reported metal–dipicolylamine modified CyD sensors, which can recognize ATP using both dipicolylamine–metal center and CyD cavities [21,22]. 

Although this kind of modified-CyD sensor captured ATP through multiple interactions, the flexibility of the ATP-complex was not enough due to the chemical bond connecting the two recognition moieties, resulting in a weak spectral change both in fluorescence and UV-Vis spectra. From this perspective, still few ATP sensors based on CyDs exist as the reaction medium for molecular recognition.

**Figure 1:**
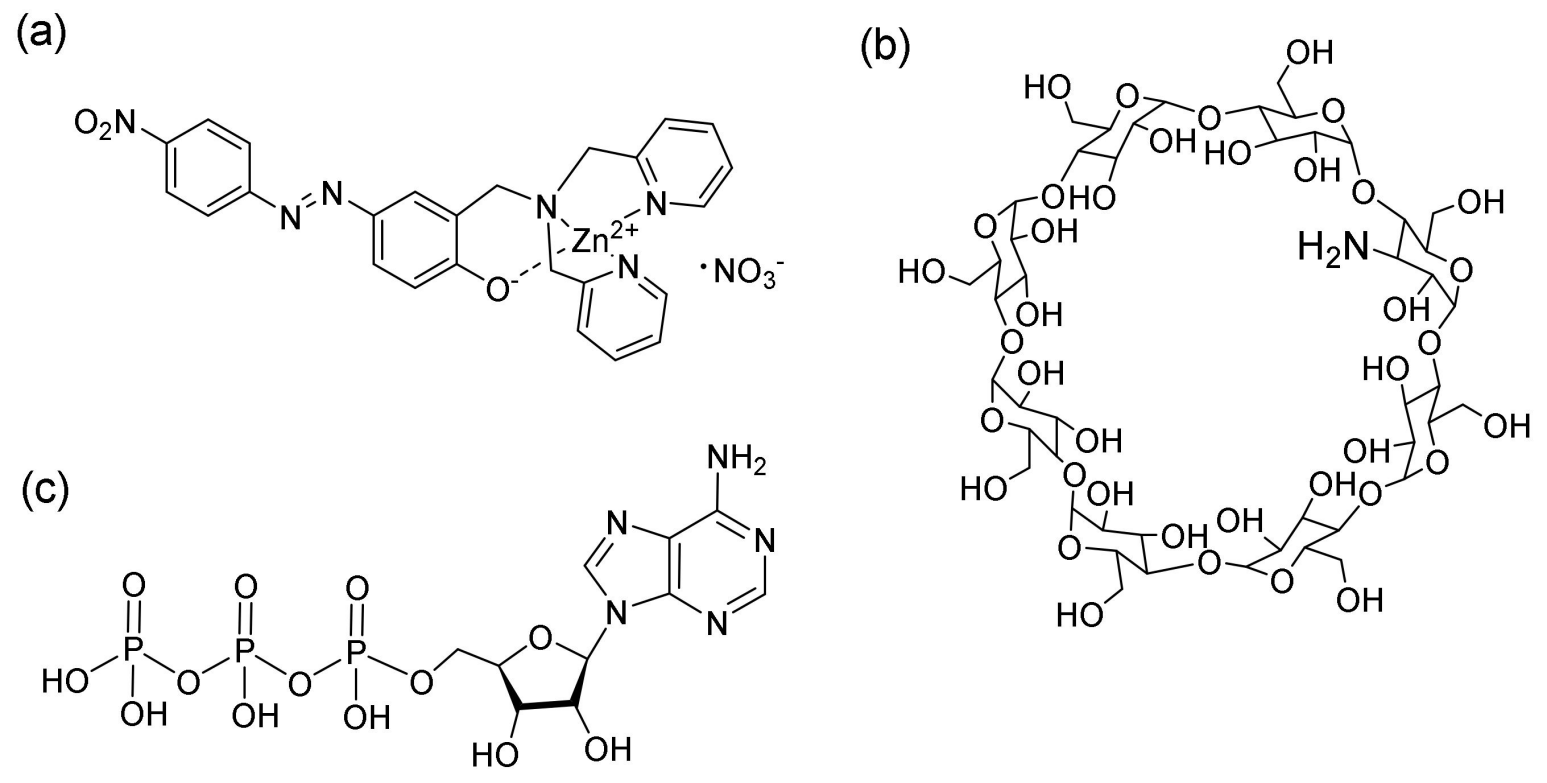
Chemical structures of (**a**) **1-Zn**, (**b**) 3-NH_2_-*γ*-CyD, and (**c**) ATP.

Herein we have designed a novel supramolecular sensing system based on the “reaction medium approach”, in which a modified CyD cavity enhances molecular recognition ability. We used Zn(II)-dipicolylamine chromophore (**1-Zn**) as the probe molecule for ATP detection. Zn(II)-dipicolylamine metal complex is an efficient unit for phosphate derivative recognition since it shows good affinity for phosphate oxyanion via metal coordination interaction [23]. We found that **1-Zn** showed UV-Vis and circular dichroism spectral changes in the presence of ATP and 3A-amino-3A-deoxy-(2A*S*,3A*S*)-*γ*-cyclodextrin (3-NH_2_-*γ*-CyD) in which an amino group is substituted for one 3-position of the glucose ring of *γ*-CyD. The structures of **1-Zn**, 3-NH_2_-*γ*-CyD, and ATP are shown in Figure 1. The ATP recognition mechanism by **1-Zn**/3-NH_2_-*γ*-CyD complex is elucidated in this study.

## 2. Results and Discussion

### 2.1. Design of **1-Zn** Complex

We synthesized ligand **1** possessing azobenzene as the chromophore by the Mannich reaction with dipicolylamine, formaldehyde, and 4-(4-nitrophenyl)azophenol. In all the experiments, the same equivalent of Zn(NO_3_)_2_ as **1** was added into an aqueous solution, and the complex of **1** with Zn(NO_3_)_2_ is shown as **1-Zn**. It is known that this type of ligand coordinates Zn(II) through the three N atoms of dipicolylamine and the O atom of the phenol moiety with high affinity (*K*_ML_ > 10^7^ M^−1^) [24,25].

### 2.2. UV-Vis Spectra

**Figure 2:**
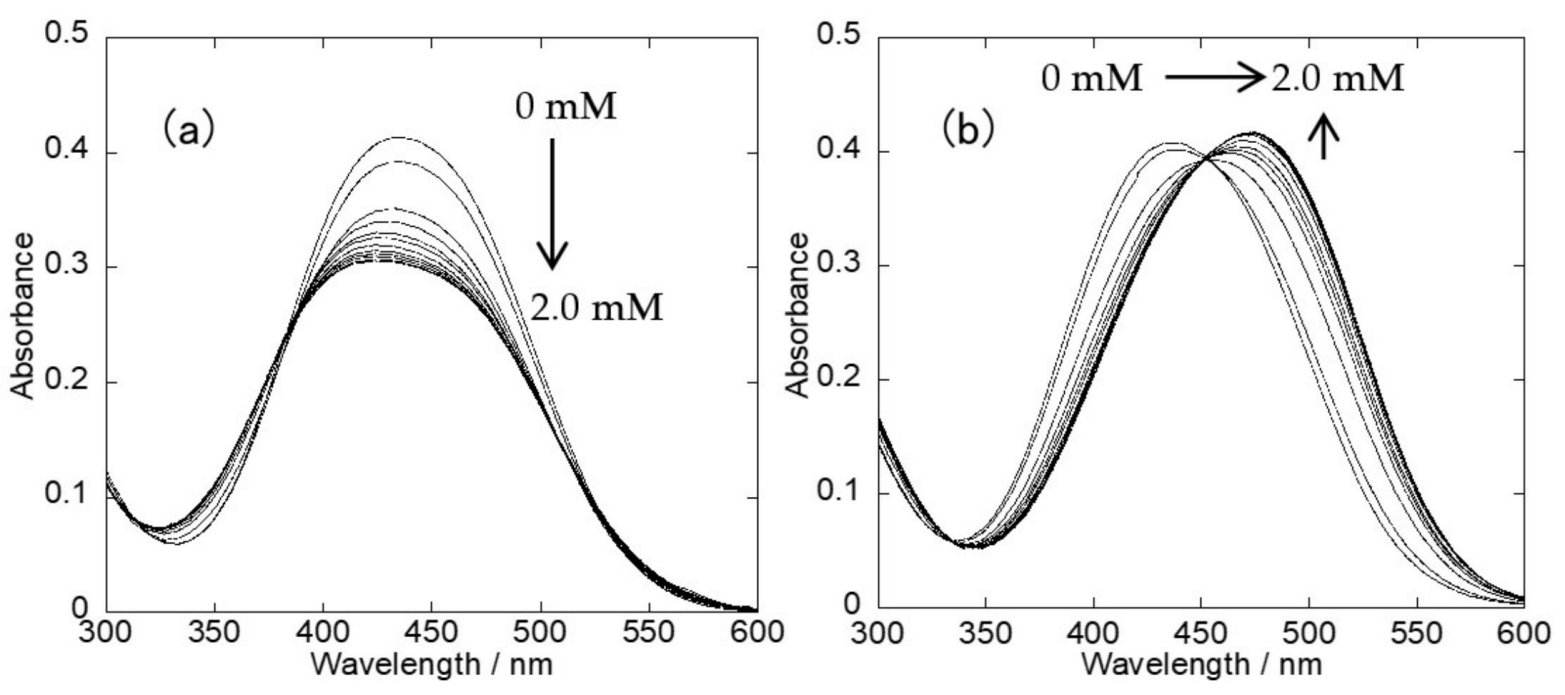
Changes in UV-Vis spectra of **1-Zn** in the (**a**) absence or (**b**) presence of 3-NH_2_-*γ*-CyD with increasing concentrations of ATP. [**1-Zn**] = 0.020 mM, [HEPES] = 5.0 mM; [3-NH_2_-*γ*-CyD] = 2.0 mM; [ATP] = 0, 0.020, 0.20, 0.40, 0.60, 0.80, 1.0, 1.2, 1.4, 1.6, 1.8, 2.0 mM, in 1% DMSO aq. at pH 7.4.

We first examined the UV-Vis spectral response of **1-Zn** upon the addition of ATP in the absence and presence of 3-NH_2_-*γ*-CyD. In the absence of 3-NH_2_-*γ*-CyD, **1-Zn** showed an absorbance maximum decrease at 434 nm with no clear peak shift, as shown in Figure 2a. In contrast, the absorbance maximum of **1-Zn** showed a redshift from 434 nm to 475 nm in the presence of 100 equivalents of 3-NH_2_-*γ*-CyD, as shown in Figure 2b. This redshift was caused by the coordination of the phosphate O atom of ATP to Zn, which weakened the O-Zn bond and increased the charge density of the phenolate O atom [24,25,26,27]. From these results, it was evident that **1-Zn** functioned as a colorimetric sensor for ATP in the presence of 3-NH_2_-*γ*-CyD.

**Figure 3:**
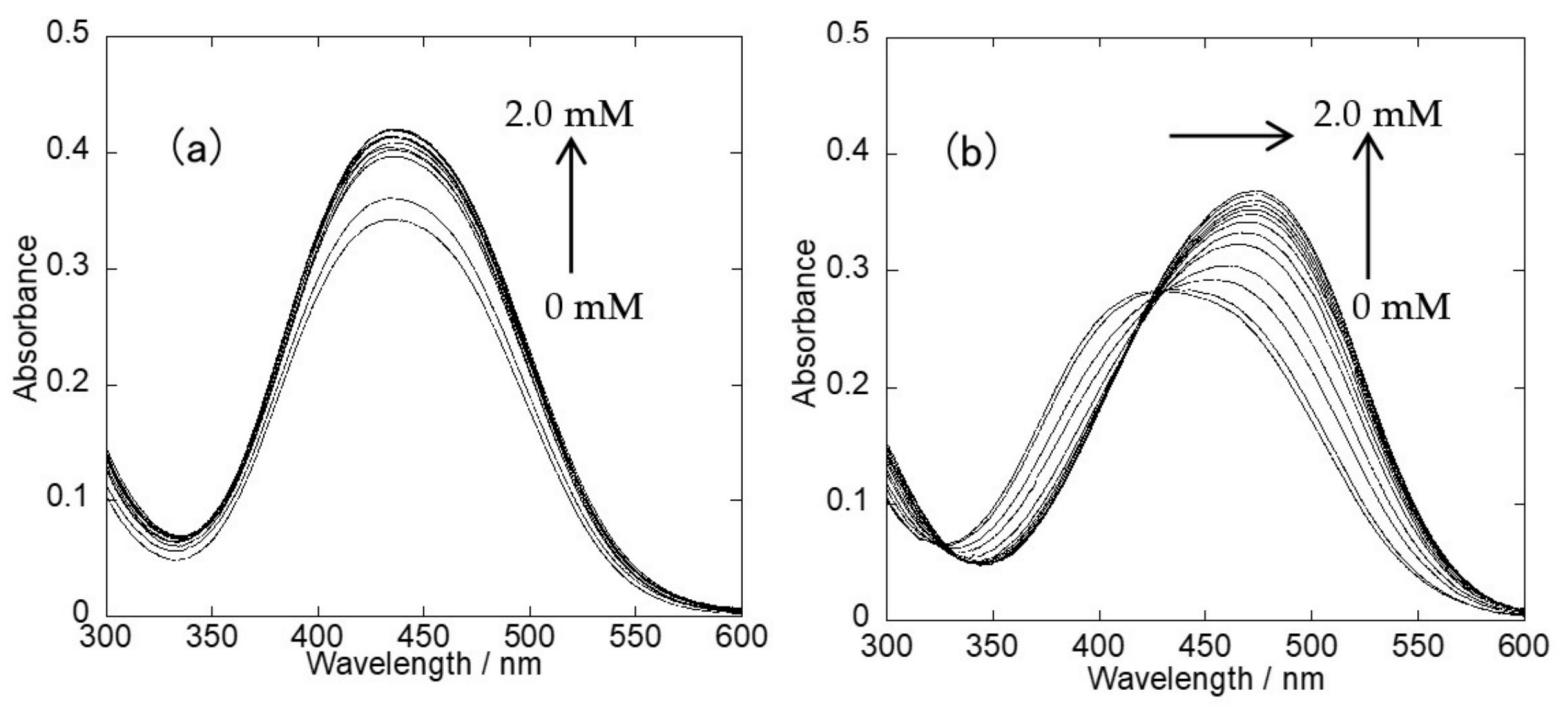
Changes in UV-Vis spectra of **1-Zn** in the (**a**) absence or (**b**) presence of ATP with increasing concentrations of 3-NH_2_-*γ*-CyD. [**1-Zn**] = 0.020 mM; [HEPES] = 5 mM; [ATP] = 2.0 mM; [3-NH_2_-*γ*-CyD] = 0, 0.020, 0.20, 0.40, 0.60, 0.80, 1.0, 1.2, 1.4, 1.6, 1.8, 2.0 mM, in 1% DMSO aq. at pH 7.4.

Afterwards, we examined the UV-Vis spectral response of **1-Zn** upon the addition of 3-NH_2_-*γ*-CyD in the absence and presence of ATP. The results are shown in Figure 3. In the absence of ATP, the addition of 3-NH_2_-*γ*-CyD increased the absorbance maximum of **1-Zn** with no clear peak shift. In the presence of ATP, the addition of 3-NH_2_-*γ*-CyD caused a redshift, as confirmed in Figure 2. These results indicated that the synergistic interaction of **1-Zn**, 3-NH_2_-*γ*-CyD, and ATP was an important factor for inducing the redshift of **1-Zn** in water.

**Figure 4:**
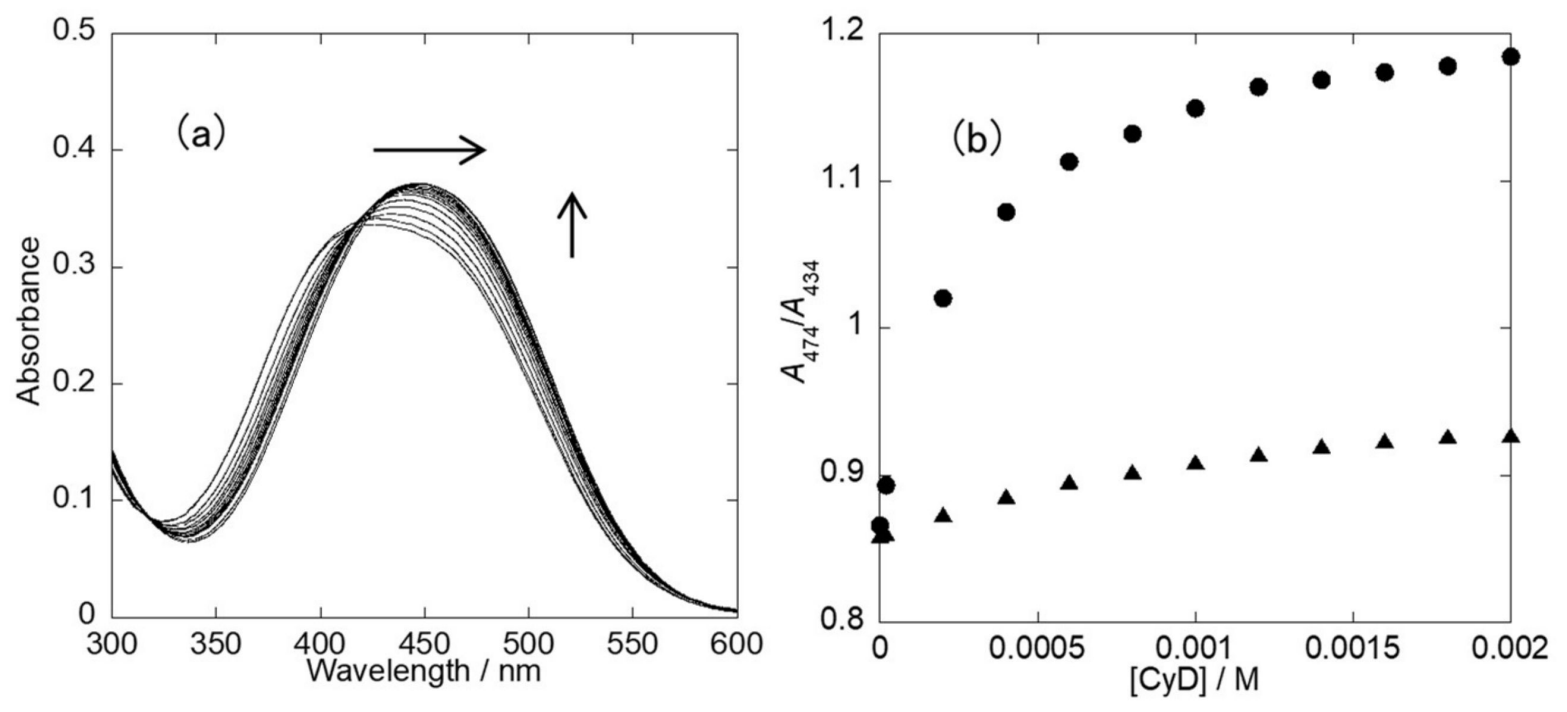
(**a**) Changes in UV-Vis spectra of **1-Zn** in the presence of ATP with increasing concentrations of *γ*-CyD. (**b**) Plot of the absorbance ratio (*A*_474_/*A*_434_) of **1-Zn** versus concentrations of (●) 3-NH_2_-*γ*-CyD and (▲) *γ*-CyD in the presence of ATP. [**1-Zn**] = 0.02 mM; [HEPES] = 5 mM; [ATP] = 2 mM; [CyD] = 0, 0.020, 0.2, 0.4, 0.6, 0.8, 1.0, 1.2, 1.4, 1.6, 1.8, 2.0 mM, in 1% DMSO aq. at pH 7.4.

To evaluate the effect of the amino group of 3-NH_2_-*γ*-CyD, *γ*-CyD titration experiments were performed. As shown in Figure 4, the addition of *γ*-CyD caused a redshift in the presence of 100 equivalents of ATP. However, it was evident from the *A*_474_/*A*_434_ radiometric plot that 3-NH_2_-*γ*-CyD exhibited more significant changes than *γ*-CyD. Furthermore, the equilibrium constants (*K*_1_, *K*_2_, and *K*_1_*K*_2_; reported in SI) were calculated by a non-linear curve fitting method on the assumption that **1-Zn** formed a 2:1 complex with 3-NH_2_-*γ*-CyD and *γ*-CyD, respectively [28]. The results are summarized in Table 1. The overall equilibrium constant (*K*_1_*K*_2_ = [HG_2_]/[H][G]^2^) of **1-Zn** and 3-NH_2_-*γ*-CyD was larger than that of **1-Zn** and *γ*-CyD. These results signified that the amino group of 3-NH_2_-*γ*-CyD played an important role in this supramolecular sensing system. 

### 2.3. Induced Circular Dichroism (ICD) Spectra

**Figure 5:**
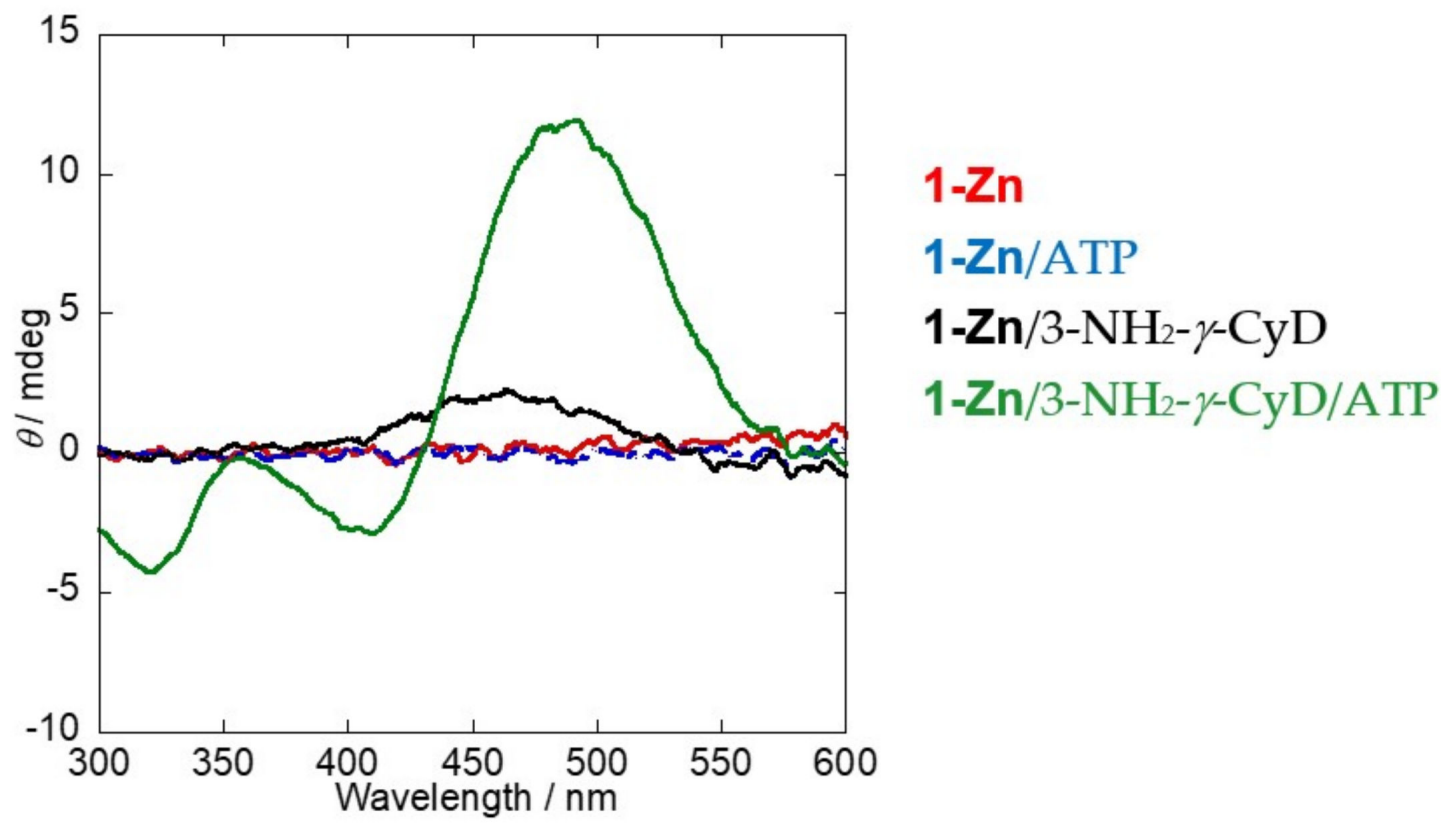
ICD spectra of **1-Zn**, **1-Zn**/ATP, **1-Zn**/3-NH_2_-*γ*-CyD, and **1-Zn**/3-NH_2_-*γ*-CyD /ATP. [**1-Zn**] = 0.040 mM, [ATP] = 4.0 mM, [3-NH_2_-*γ*-CyD] = 4.0 mM, [HEPES] = 5.0 mM, in 2% DMSO aq. at pH 7.4.

To obtain the structural information of the supramolecular **1-Zn**/3-NH_2_-*γ*-CyD/ATP complex, ICD spectra measurements were carried out. When a chiral cyclodextrin cavity interacts with an achiral azobenzene (= **1-Zn**) by forming an inclusion complex, the ICD signal is observed at the absorption band of **1-Zn**. From this ICD spectral change, it is possible to estimate the inclusion of a **1-Zn** dimer inside *γ*-CyD, which induces a change in the UV-Vis spectra [29,30,31]. As shown in Figure 5, **1-Zn** and **1-Zn**/ATP did not produce any ICD spectral response since their structures are achiral. In the case of **1-Zn**/3-NH_2_-*γ*-CyD, a slight change in ICD spectra was noted in the wavelength range from 400 nm to 530 nm. This means that 3-NH_2_-*γ*-CyD interacts with **1-Zn** because of its chiral nature. On the other hand, **1-Zn**/3-NH_2_-*γ*-CyD/ATP showed a much stronger ICD spectral response than **1-Zn**/3-NH_2_-*γ*-CyD. These results demonstrated that ATP complexation fixed the location of **1-Zn** in the 3-NH_2_-*γ*-CyD cavity. To determine the stoichiometry of **1-Zn**, 3-NH_2_-*γ*-CyD, and ATP, the continuous variation method was employed. The Job plots shown in Figure S1 indicated that the stoichiometry of **1-Zn** and ATP resulted to be 2:1 (Figure S1a), and that of **1-Zn** and 3-NH_2_-*γ*-CyD to be 2:1 (Figure S1b). Therefore, the total stoichiometry of **1-Zn**/3-NH_2_-*γ*-CyD/ATP complex was determined to be 2:1:1.

**Figure 6:**
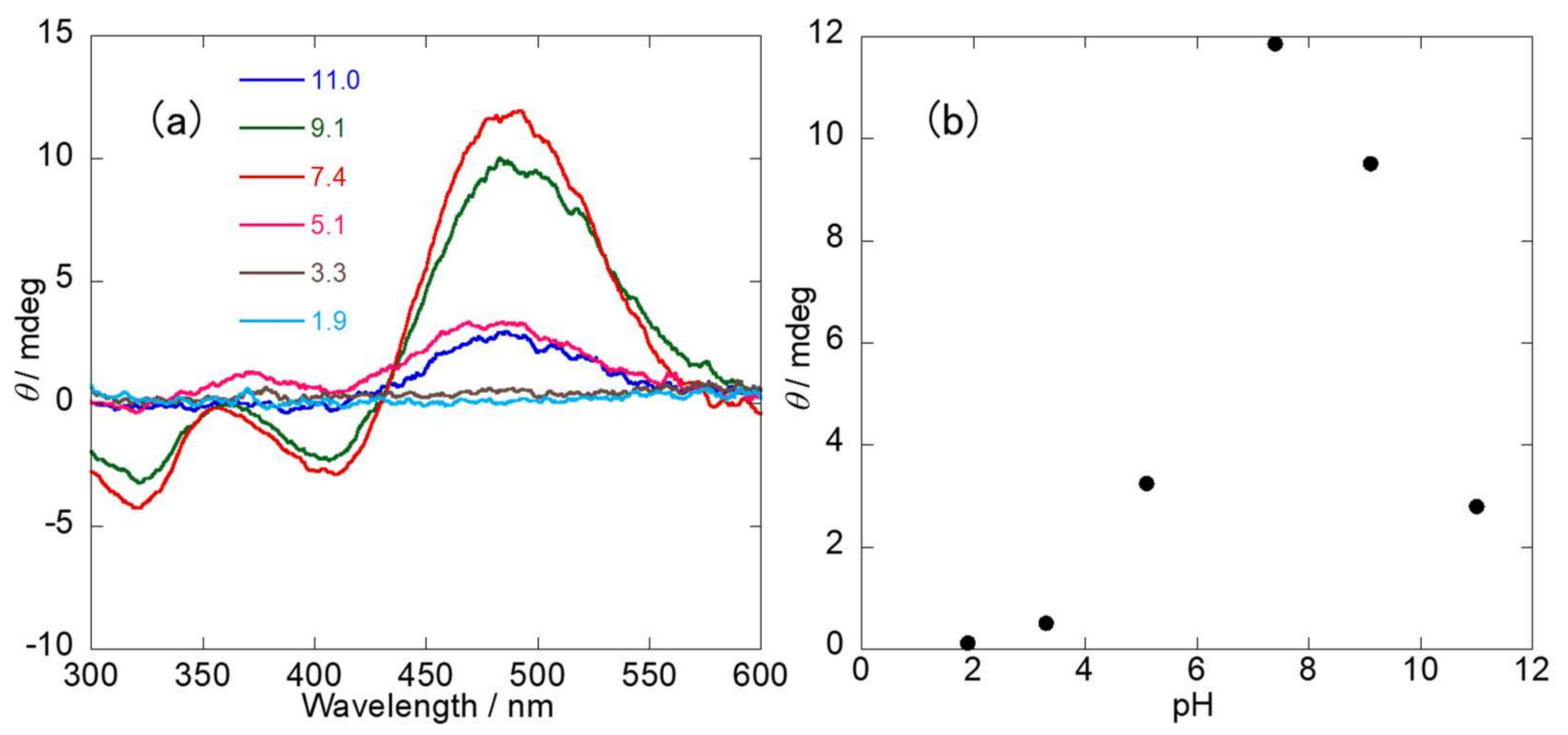
(**a**) ICD spectra of **1-Zn**/3-NH_2_-*γ*-CyD/ATP at different pH values. (**b**) Plot of *θ*_490_—of **1-Zn**/3-NH_2_-*γ*-CyD /ATP versus pH. [**1-Zn**] = 0.040 mM, [ATP] = 4.0 mM, [3-NH_2_-*γ*-CyD] = 4.0 mM, in 2% DMSO aq.

We also examined the pH effect on the **1-Zn**/3-NH_2_-*γ*-CyD/ATP complex. Figure 6 shows the ICD spectra obtained under different pH conditions. The strongest ICD spectral response was obtained at the neutral pH of 7.4, suggesting that the **1-Zn**/3-NH_2_-*γ*-CyD/ATP complex was most stable at pH 7.4. When the pH was decreased to 5.1, the number of deprotonated phosphate OH groups in ATP decreased as well. This is confirmed by the p*K*_a_ of the secondary phosphate of ATP (p*K*_a_ = 6.50) as reported [32]. This means that electrical neutrality of the **1-Zn**/3-NH_2_-*γ*-CyD/ATP system could not be maintained in acidic conditions. Further decreasing the pH from 5.1 to an even lower value increased the number of protonated azo groups. Thus, **1-Zn** became more hydrophilic, resulting into the dissociation of the **1-Zn** and 3-NH_2_-*γ*-CyD complex. On the other hand, the increase in pH from 7.4 to 11.0 produced a decrease in the number of protonated amino groups of 3-NH_2_-*γ*-CyD. The correspondent p*K*_a_ of 3-NH_2_*-γ*-CyD is reported to be 7.73 [33], and therefore, a similar value is expected for the **1-Zn**/3-NH_2_-*γ*-CyD complex. Similarly to acidic conditions, the increase of pH to 11 produced a loss of electrical neutrality as well, resulting in a lower ICD spectral response.

### 2.4. NMR Spectra

**Figure 7:**
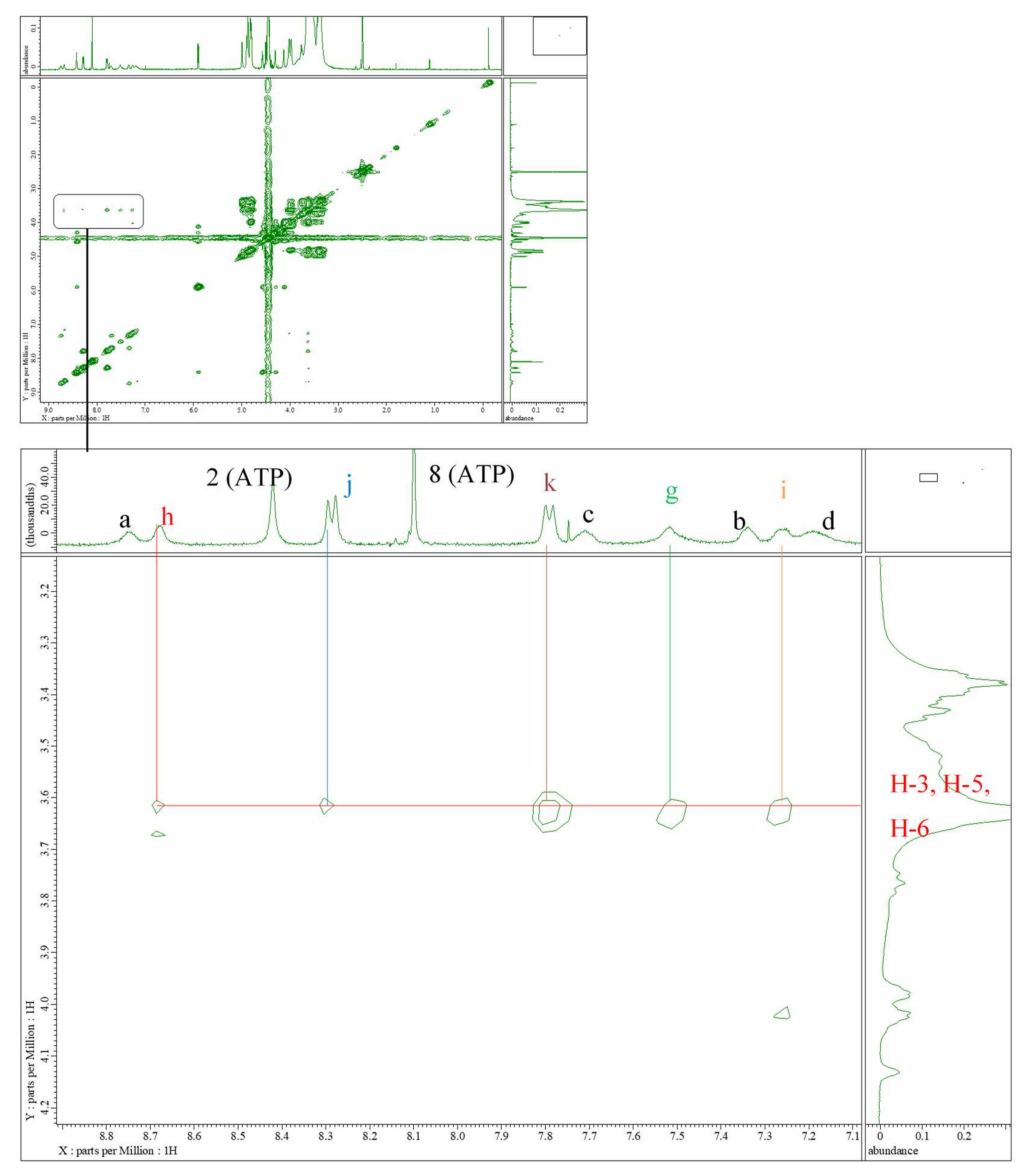
NOESY spectrum of 1-Zn/3-NH_2_-*γ*-CyD/ATP (22 ℃, mixing time: 0.5 s, 160 scans).

**Scheme 1:**
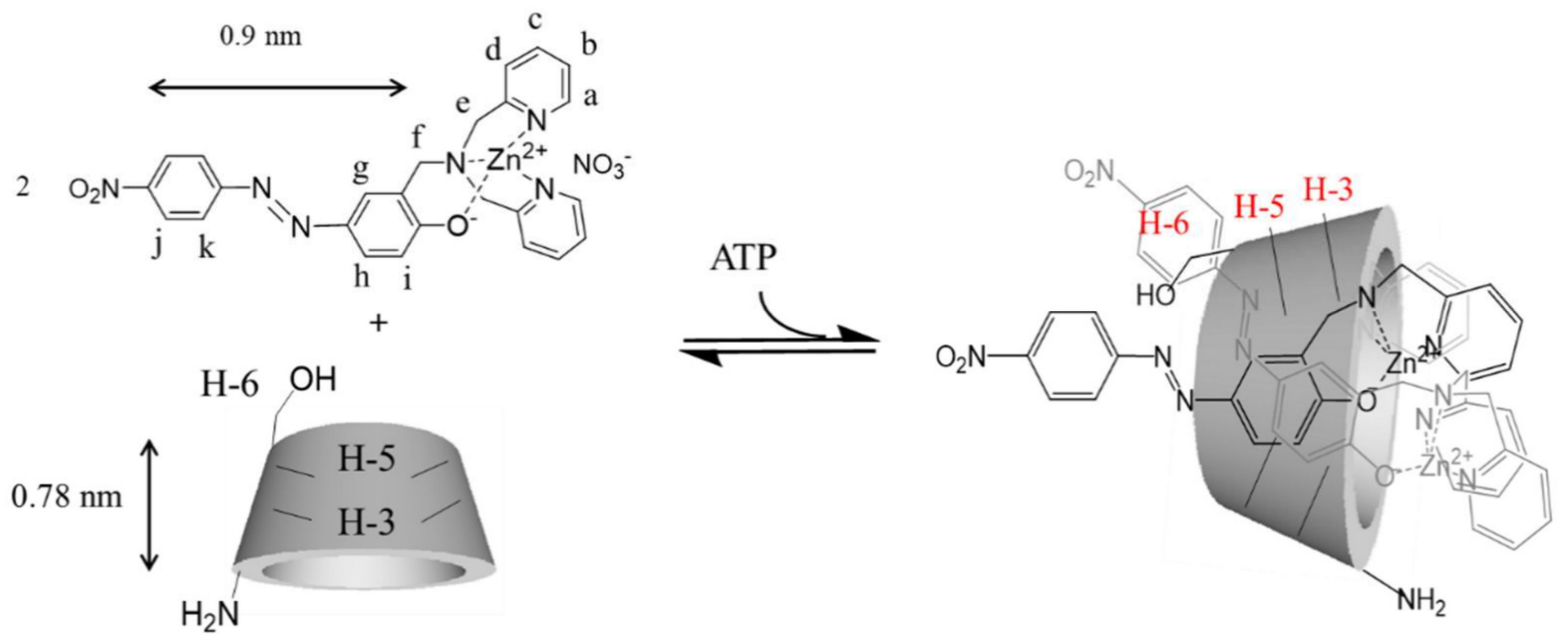
Suggested conformation of 1-Zn and 3-NH_2_-*γ*-CyD in the presence of ATP.

To obtain the detailed structure of the inclusion complex, a NOESY experiment of **1-Zn**/3-NH_2_-*γ*-CyD/ATP was carried out. Figure 7 shows a NOESY spectrum for **1-Zn**/3-NH_2_-*γ*-CyD/ATP. NOE correlation peaks were observed between the protons of the azobenzene moiety of **1-Zn** (g, h, i, j, k) and the protons of H-3, H-5, and H-6 of 3-NH_2_-*γ*-CyD. The azobenzene peaks in the NOESY spectrum were assigned by COSY (data shown in Figure S2). The cavity depth of 3-NH_2_-*γ*-CyD is 0.78 nm and the molecular length of *trans*-azobenzene is 0.9 nm [34]. Based on this information, we estimated that the phenol moiety of **1-Zn** was localized in the 3-NH_2_-*γ*-CyD cavity, and the nitrophenyl moiety was situated near H-6 of 3-NH_2_-*γ*-CyD, as shown in Scheme 1.

### 2.5. Supramolecular Structure

**Scheme 2:**
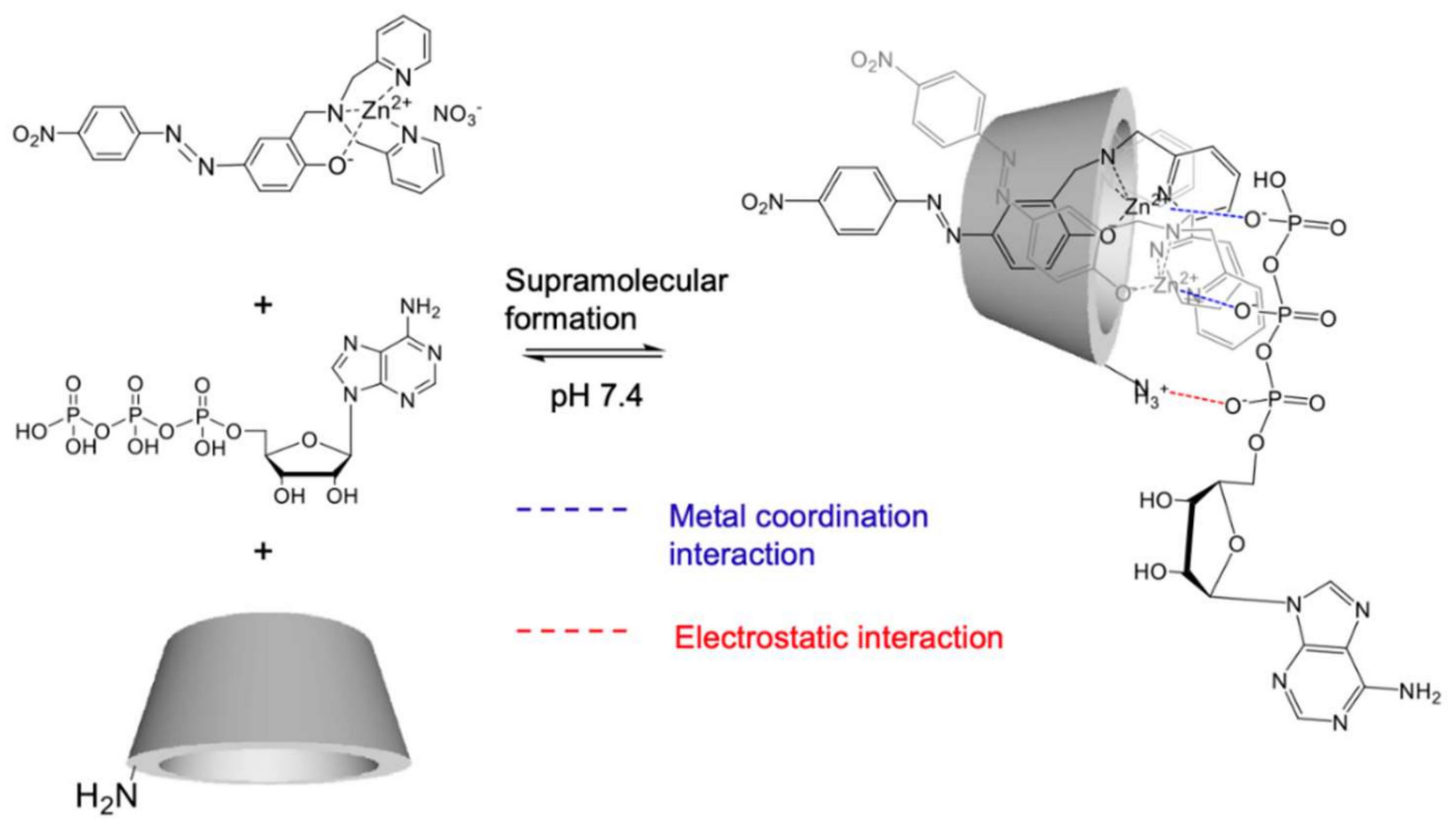
Suggested supramolecular structure of 1-Zn/3-NH_2_-*γ*-CyD/ATP.

Based on all above results in Section 2.2, Section 2.3 and Section 2.4, a possible structure of the supramolecular complex is shown in Scheme 2. Two molecules of **1-Zn** are included in 3-NH_2_-*γ*-CyD’s cavity, two phosphate oxyanions of ATP coordinate with **1-Zn**, and one oxyanion interacts with the NH_3_^+^ group of 3-NH_2_-*γ*-CyD via electrostatic interaction at pH 7.4.

### 2.6. Effect of Other Phosphate Derivatives

To assess the **1-Zn**/3-NH_2_-*γ*-CyD sensing system by using other phosphate derivatives, we examined changes in the UV-Vis and ICD spectral response upon the addition of ADP, AMP, triphosphate (Tri), pyrophosphate (PPi), and phosphate (Pi). From the results in Figures S3 and S4, similar UV-Vis and ICD spectral responses by ATP addition were observed upon the addition of oligophosphate derivatives (ADP, Tri, and PPi), but not monophosphates (Pi and AMP). These results suggested that at least two phosphate units were necessary to form the supramolecular structure. Furthermore, it was evident that the interactions involved just the phosphate oxyanion moiety of ATP, and not the ribose or adenine one, as shown in Scheme 2.

## 3. Experimental Section

### 3.1. Reagents

All chemical reagents were purchased from commercial suppliers (Sigma Aldrich, Tokyo Chemical Industry (TCI), Kanto Chemical, or FUJIFILM Wako Chemicals) and used without further purification unless otherwise stated. Adenosine-5′-triphosphate (ATP) was purchased from TCI as disodium salt hydrate. 3A-amino-3A-deoxy-(2A*S*,3A*S*)-*γ*-cyclodextrin (3-NH_2_-*γ*-CyD) was purchased from TCI as hydrate. Gamma-cyclodextrin (*γ*-CyD) and 3-NH_2_-*γ*-CyD were used after drying under vacuum. The concentration of stock Zn(NO_3_)_2_ aqueous solution was determined by inductively coupled plasma atomic emission spectroscopy (SPS3500DD, Hitachi High-Tech Science Corporation) and by a calibration curve created by measuring calibration solutions with different concentrations prepared from zinc standard solution (1000 mg/L, FUJIFILM Wako Chemicals, NOsaka, Japan).

### 3.2. Synthesis of 1 (2-Dipicolylaminomethyl-4-(4-nitrophenylazo)phenol)

A mixture of dipicolylamine (0.319 g, 1.60 mmol) and 37% formaldehyde aqueous solution (0.421 g, 5.19 mmol) in 5 mL methanol was refluxed for 2 h. A solution of 4-(4-nitrophenyl)azophenol (0.371 g, 1.53 mmol) in 20 mL methanol was added and the reaction mixture was refluxed for 19 h. After refluxing, the reaction mixture was cooled to room temperature and filtered. The crude product was initially purified by column chromatography on silica gel (Merck Silica gel 60 for column chromatography, eluent: CH_2_Cl_2_:methanol = 100:6) and further purified by HPLC (LC-918, Japan Analytical Industry, column: JAIGEL-2H, mobile phase: CHCl_3_). UV-Vis detection was performed at 254 nm at a flow rate of 3.8 mL min^-1^. After removal of the solvent, a red solid was obtained (yield: 173.8 mg, 25%).

^1^H NMR (300 MHz, CDCl_3_) *δ*: 8.58 (2H, dd, *J* = 5.0, 0.9 Hz), 8.35 (2H, d, *J* = 9.0 Hz), 7.95 (2H, d, *J* = 9.0 Hz), 7.90 (1H, dd, *J* = 8.7, 2.5 Hz), 7.80 (1H, d, *J* = 2.5 Hz), 7.65 (2H, td, *J* = 7.7, 1.7 Hz), 7.34 (2H, d, *J* = 8.0 Hz), 7.19 (2H, ddd, *J* = 7.6, 5.0, 0.8 Hz), 7.06 (1H, d, *J* = 8.7 Hz), 3.96 (4H, s), 3.91 (2H, s). HR-FAB-MS(+): Calcd for C_25_H_22_N_6_O_3_ [M+H]^+^, 455.1826, Found, 455.1782. Anal. Calcd for C_25_H_22_N_6_O_3_: C, 66.07; H, 4.88; N, 18.46. +0.55H_2_O: C, 64.65; H, 5.02; N, 18.09. Found: C, 64.61; H.4.69; N, 17.78.

### 3.3. Measurement Instruments

UV-Vis spectra were recorded using a JASCO V-560 spectrometer with a 10-mm quartz cell. CD spectra were recorded using a JASCO J-820 spectropolarimeter with a 10-mm quartz cell and a Peltier thermocontroller (JASCO Co., Tokyo, Japan). NMR spectra were recorded with a JEOL JNM-ECX 300 spectrometer or a JEOL JNM-ECA 500 spectrometer (JEOL Ltd., Tokyo, Japan) using 5-mm NMR tubes at room temperature. Stock solutions of **1** and Zn(NO_3_)_2_ were prepared by adding DMSO-*d*_6_ and D_2_O, respectively. Stock solutions of 3-NH_2_-*γ*-CyD and ATP were prepared by adding D_2_O, and their pD was adjusted to 7.4 by adding NaOD or DCl. The pD value was calculated as follows: pD = pH_obs_ + 0.4, [35] where pH_obs_ is the observed pH value obtained by a pH meter. Sample solution was prepared by adding stock solutions of **1**, Zn(NO_3_)_2_, 3-NH_2_-*γ*-CyD, and ATP.

## 4. Conclusions

We have developed a novel **1-Zn**/3-NH_2_-*γ*-CyD/ATP supramolecular sensing system and clarified its supramolecular structure by UV-Vis, ICD, and NMR measurements. **1-Zn** showed a redshift in the UV-Vis spectra and strong ICD spectral response in the presence of both 3-NH_2_-*γ*-CyD and ATP. The Job plots of the ICD spectral response revealed that the stoichiometry of **1-Zn**/3-NH_2_-*γ*-CyD/ATP complex is 2:1:1. The NOESY spectrum suggested that **1-Zn** is localized in the 3-NH_2_-*γ*-CyD cavity in the presence of ATP. However, the **1-Zn**/3-NH_2_-*γ*-CyD host system was not specific to ATP because it responded to other phosphate derivatives having more than one phosphate unit. It is evident that the obtained structural information is very important for the design of supramolecular sensing systems for ATP, with high selectivity and sensitivity in water.

## Figures and Tables

**Table 1:** Equilibrium constants of **1-Zn**/CyD complexes in the presence of ATP.

	*K*_1_/M^−1^	*K*_2_/M^−1^	*K*_1_*K*_2_/M^−2^
*γ*-CyD	1.95 × 10^3^	1.02 × 10^3^	1.99 × 10^6^
3-NH_2_-*γ*-CyD	2.64 × 10^3^	1.37 × 10^3^	3.62 × 10^6^

## Data Availability

Not applicable.

## Supplementary Materials

The following are available online at https://www.mdpi.com/article/10.3390/ijms22094683/s1: Section 1: Calculation of binding constant of (**1-Zn**)_2_/3-NH_2_-*γ*-CyD (or *γ*-CyD) complex, Figure S1: Job plots of the ICD spectral response, Figure S2: H-H COSY spectrum of **1-Zn**/3-NH_2_-*γ*-CyD/ATP, Figure S3 and S4: changes in UV-Vis/ICD spectra and wavelength shifts of **1-Zn** upon the addition of phosphate derivatives in the presence of 3-NH_2_-*γ*-CyD, and Figure S5: 1H NMR spectrum of **1**, Figure S6: FAB-Mass spectrum of **1**.

## References

[1] Lehn J.M. (1995). Supramolecular Chemistry: Concepts and Perspectives.

[2] Pedersen C.J. (1967). Cyclic polyethers and their complexes with metal salts. J. Am. Chem. Soc..

[3] Fischer E. (1894). Einfluss der configuration auf die wirkung der enzyme. II. Ber. Dtsch. Chem. Ges..

[4] Matsushita A., Kuwabara T., Nakamura A., Ikeda H., Ueno A. (1997). Guest-induced color change and molecule-sensing ability of *p*-nitrophenol-modified cyclodextrins. J. Chem. Soc. Perkin Trans. 2.

[5] Kuwabara T., Shiba K., Nakajima H., Ozawa M., Miyajima N., Hosoda M., Kuramoto N., Suzuki Y. (2006). Host-guest complexation affected by pH and length of spacer for hydroxyazobenzene-modified cyclodextrins. J. Phys. Chem. A.

[6] Kuwabara T., Sugiyama K. (2013). Hyperchromisity and molecular recognition of a novel modified *β*-cyclodextrin tethering with phenylaminoazobenzene. Anal. Sci..

[7] Zhang S., Shi Y., Wanga W., Yuan Z. (2015). Study on the association between CTD peptides and zinc(ii)-dipicolylamine appended *β*-cyclodextrin. RSC Adv..

[8] Yadav V.B., Rai P., Sagir H., Kumar A., Siddiqui I.R. (2018). A green route for the synthesis of pyrrolo[2,3-*d*]pyrimidine derivatives catalyzed by *β*-cyclodextrin. New. J. Chem..

[9] Breslowong R., Dong S.D. (1998). Biomimetic reactions catalyzed by cyclodextrins and their derivatives. Chem. Rev..

[10] Shimpuku C., Ozawa R., Sasaki A., Sato F., Hashimoto T., Yamauchi A., Suzuki I., Hayashita T. (2009). Selective glucose recognition by boronic acid azoprobe/*γ*-cyclodextrin complexes in water. Chem. Commun..

[11] Kumai M., Kozuka S., Hashimoto T., Hayashita T. (2010). Design of boronic acid fluorophore/aminated cyclodextrin complexes for sugar sensing in water. J. Ion Exch..

[12] Kumai M., Kozuka S., Samizo M., Hashimoto T., Suzuki I., Hayashita T. (2012). Glucose recognition by a supramolecular complex of boronic acid fluorophore with boronic acid-modified cyclodextrin in water. Anal. Sci..

[13] Sugita K., Tsuchido Y., Kasahara C., Casulli M.A., Fujiwara S., Hashimoto T., Hayashita T. (2019). Selective sugar recognition by anthracene-type boronic acid fluorophore/cyclodextrin supramolecular complex under physiological pH condition. Front. Chem..

[14] Azath I.A., Suresh P., Pitchumani K. (2011). Per-6-ammonium-*β*-cyclodextrin/*p*-nitrophenol complex as a colorimetric sensor for phosphate and pyrophosphate anions in water. Sens. Actuators B.

[15] Mahato P., Ghosh A., Mishra S.K., Shrivastav A., Mishra S., Das A. (2011). Zn(II)-cyclam based chromogenic sensors for recognition of ATP in aqueous solution under physiological conditions and their application as viable staining agents for microorganism. Inorg. Chem..

[16] Yang N., Wei Y., Kang X., Su Z. (2015). Selective recognition and sensing for adenosine triphosphate by label-free electrochemistry based on its inclusion with per-6-ammonium-*β*-cyclodextrin. Anal. Methods.

[17] Hu T., Na W., Yan X., Su X. (2017). Sensitive fluorescence detection of ATP based on host-guest recognition between near-infrared *β*-cyclodextrin-CuInS_2_ QDs and aptamer. Talanta.

[18] Song C., Xiao Y., Li K., Zhang X., Lu Y. (2019). *β*-Cyclodextrin polymer based fluorescence enhancement method for sensitive adenosine triphosphate detection. Chin. Chem. Lett..

[19] Kanagaraj K., Xiao C., Rao M., Fan C., Borovkov V., Cheng G., Zhou D., Zhong Z., Su D., Yu X. (2020). A quinoline-appended cyclodextrin derivative as a highly selective receptor and colorimetric probe for nucleotides. iScience.

[20] Singh V.R., Singh P.K. (2020). A supramolecule based fluorescence turn-on and ratiometric sensor for ATP in aqueous solution. J. Mater. Chem. B.

[21] Fujita K., Fujiwara S., Yamada T., Tsuchido Y., Hashimoto T., Hayashita T. (2017). Design and Function of supramolecular recognition systems based on guest-targeting probe-modified cyclodextrin receptors for ATP. J. Org. Chem..

[22] Yamada T., Fujita K., Fujiwara S., Tsuchido Y., Hashimoto T., Hayashita T. (2018). Development of dipicolylamine-modified cyclodextrins for the design of selective guest-responsive receptors for ATP. Molecules.

[23] Ngo H.T., Liu X., Jolliffe K.A. (2012). Anion recognition and sensing with Zn(II)–dipicolylamine complexes. Chem. Soc. Rev..

[24] Lee D.H., Im J.H., Son S.U., Chung Y.K., Hong J.I. (2003). An azophenol-based chromogenic pyrophosphate sensor in water. J. Am. Chem. Soc..

[25] Lee D.H., Kim S.Y., Hong J.I. (2004). A fluorescent pyrophosphate sensor with high selectivity over ATP in water. Angew. Chem. Int. Ed..

[26] Roy B., Rao A.S., Ahn K.H. (2011). Mononuclear Zn(II)- and Cu(II)-complexes of a hydroxynaphthalene-derived dipicolylamine: Fluorescent sensing behaviors toward pyrophosphate ions. Org. Biomol. Chem..

[27] Yang S., Feng G., Williams N.H. (2012). Highly selective colorimetric sensing pyrophosphate in water by a NBD-phenoxo-bridged dinuclear Zn(II) complex. Org. Biomol. Chem..

[28] Hargrove A.E., Zhong Z., Sessler J.L., Anslyn E.V. (2010). Algorithms for the determination of binding constants and enantiomeric excess in complex host: Guest equilibria using optical measurements. New J. Chem..

[29] Mayer B., Zhang X., Nau W.M., Marconi G. (2001). Co-conformational variability of cyclodextrin complexes studied by induced circular dichroism of azoalkanes. J. Am. Chem. Soc..

[30] Kodaka M. (1993). A general rule for circular dichromism induced by a chiral macrocycle. J. Am. Chem. Soc..

[31] Tinoco I. (1962). Theoretical aspects of optical activity. Part two: Polymers. Adv. Chem. Phys..

[32] Dean J.A. (1985). Lange’s Handbook of Chemistry.

[33] Takahashi K., Andou K., Fujiwara S. (2012). Characterization and structural determination of 3A-amino-3A-deoxy-(2AS, 3AS)-cyclodextrins by NMR spectroscopy. Polym. J..

[34] Huang C., Lv J., Tian X., Wang Y., Yu Y., Liu J. (2015). Miniaturized swimming soft robot with complex movement actuated and controlled by remote light signals. Sci. Rep..

[35] Glasoe P.K., Long F.A. (1960). Use of glass electrodes to measure acidities in deuterium oxide. J. Phys. Chem..

