# Supplementary material for: Supramolecular Zn(II)-Dipicolylamine-Azobenzene-Aminocyclodextrin-ATP Complex: Design and ATP Recognition in Water
Source: Int J Mol Sci. 2021 Apr 28;22(9):4683. doi: 10.3390/ijms22094683 (PMC8125763; doi:10.3390/ijms22094683)
Supplement: Supplementary file 1 [file ijms-22-04683-s001.zip › ijms-1182113-supplementary.pdf]

Electronic Supplementary Information

Supramolecular Zn(II)-Dipicolylamine-Azobenzene-Aminocyclodextrin-ATP Complex: Design and ATP Recognition in Water

Shohei MINAGAWA,* Shoji FUJIWARA,*,**
Takeshi HASHIMOTO,*† and Takashi HAYASHITA*†

** Department of Materials and Life Sciences, Faculty of Science and Technology, Sophia University, 7-1 Kioi-cho, Chiyoda-ku, Tokyo 102-8554, Japan. Fax: +81-3-3238-3361; Tel: +81-3-3238-3372; E-mail: t-hasimo@sophia.ac.jp, ta-hayas@sophia.ac.jp*

*** Department of Material and Life Chemistry, Faculty of Engineering, Kanagawa University, 3-27-1 Rokkakubashi, Kanagawa-ku, Yokohama-shi, Kanagawa 221-8686, Japan*

Contents

1. **Calculation of Binding Constant of (1-Zn)$_2$/3-NH$_2$-γ-CyD (or γ-CyD) complex** **3**

2. **Job plots of ICD spectral responses** **5**
   Figure S1
   (a) Job plots of ICD spectral response of **1-Zn** and ATP in the presence of 3-NH$_2$-γ-CyD.
   (b) Job plots of ICD spectral response of **1-Zn** and 3-NH$_2$-γ-CyD in the presence of ATP.

3. **H-H COSY spectrum** **6**
   Figure S2
   H-H COSY spectrum of **1-Zn**/3-NH$_2$-γ-CyD/ATP.

4. **Spectral Changes of 1-Zn with addition of 3-NH$_2$-γ-CyD** **7**
   Figure S3
   (a) Changes in UV-Vis spectra and (b) wavelength shifts (from 435.5 nm) of **1-Zn** upon addition of phosphate derivatives in the presence of 3-NH$_2$-γ-CyD.
   Figure S4
   (a) Changes in ICD spectra and (b) changes in ICD intensity ($\Delta\theta_{490}$) of **1-Zn** upon addition of phosphate derivatives in the presence of 3-NH$_2$-γ-CyD.

5. **Identification of 1** **8**
   Figure S5
   ^{1}H NMR spectrum of **1** (solvent: $CDCl_3$).
   Figure S6
   FAB-Mass spectrum of **1**

1. Calculation of Binding Constant of (1-Zn)$_2$/3-NH$_2$-γ-CyD (or γ-CyD) complex

When CyDs (H) and **1-Zn** (G) form 1:2 complexes, the equilibrium equations can be represented by eqs. (1) and (2):

$$\mathrm{H} + \mathrm{G} \rightleftharpoons \mathrm{HG} \tag{1}$$

$$\mathrm{HG} + \mathrm{G} \rightleftharpoons \mathrm{HG_2} \tag{2}$$

The equilibrium constants for each equilibrium equation are defined by eq. (3) and (4), respectively,

$$K_1 = \frac{[\mathrm{HG}]}{[\mathrm{H}][\mathrm{G}]} \tag{3}$$

$$K_2 = \frac{[\mathrm{HG_2}]}{[\mathrm{HG}][\mathrm{G}]} \tag{4}$$

Assigning the total concentrations of H and G as $[\mathrm{H}]_t$ and $[\mathrm{G}]_t$, respectively, gives mass balance eq. (5) and (6):

$$[\mathrm{H}]_t = [\mathrm{H}] + [\mathrm{HG}] + [\mathrm{HG_2}] \tag{5}$$

$$[\mathrm{G}]_t = [\mathrm{G}] + [\mathrm{HG}] + 2[\mathrm{HG_2}] \tag{6}$$

Using eqs.(3)-(6), eqs.(7)-(9) are given as follows,

$$[\mathrm{H}] = \frac{[\mathrm{H}]_\mathrm{t}}{1 + K_1[\mathrm{G}] + K_1K_2[\mathrm{G}]^2} \tag{7}$$

$$[\mathrm{HG}] = \frac{K_1[\mathrm{G}][\mathrm{H}]_\mathrm{t}}{1 + K_1[\mathrm{G}] + K_1K_2[\mathrm{G}]^2} \tag{8}$$

$$[\mathrm{HG_2}] = \frac{K_1K_2[\mathrm{G}]^2[\mathrm{H}]_\mathrm{t}}{1 + K_1[\mathrm{G}] + K_1K_2[\mathrm{G}]^2} \tag{9}$$

Substituting eqs.(8) and (9) into eq.(6), yields eq.(10):

$$[\mathrm{G}]_\mathrm{t} = [\mathrm{G}] + \frac{K_1[\mathrm{G}] + 2K_1K_2[\mathrm{G}]^2}{1 + K_1[\mathrm{G}] + K_1K_2[\mathrm{G}]^2}[\mathrm{H}]_\mathrm{t} \tag{10}$$

Rearranging eq. (10) results in a cubic equation for [G] as eq.(11):

$$K_1K_2[\mathrm{G}]^3 + (K_1 + 2K_1K_2[\mathrm{H}]_\mathrm{t} - K_1K_2[\mathrm{G}]_\mathrm{t}) + (1 + K_1[\mathrm{H}]_\mathrm{t} - K_1[\mathrm{G}]_\mathrm{t}) - [\mathrm{G}]_\mathrm{t} \tag{11}$$

Absorbance A can be described as eq.(12) from Lambert-Beer's law,

$$A = d\left(\varepsilon_\mathrm{H}[\mathrm{H}] + \varepsilon_\mathrm{G}[\mathrm{G}] + \varepsilon_\mathrm{HG}[\mathrm{HG}] + \varepsilon_{\mathrm{HG_2}}[\mathrm{HG_2}]\right) \tag{12}$$

Since CyD has no absoprbance in the absorbance range of **1-Zn**, ε_H is 0. In this study d is 1 cm. Applying these values to eq. (12) gives eq. (13):

$$A = \varepsilon_\mathrm{G}[\mathrm{G}] + \varepsilon_\mathrm{HG}[\mathrm{HG}] + \varepsilon_{\mathrm{HG_2}}[\mathrm{HG_2}] \tag{13}$$

Here we define A_0 as the absorbance of **1-Zn** without CyD, and A_0 can be expressed as eq. (14),

$$A_0 = \varepsilon_G[\mathrm{G}] \tag{14}$$

When we define ΔA as eq. (15), eq. (16) is finnaly obtained from eqs. (13) and (14),

$$\Delta A = A - A_0 \tag{15}$$

$$\Delta A = \frac{[\mathrm{H}]_\mathrm{t}\{K_1 \varepsilon_{\mathrm{HG}}[\mathrm{G}] + K_1 K_2 \varepsilon_{\mathrm{HG_2}}[\mathrm{G}]^2\}}{1 + K_1[\mathrm{G}] + K_1 K_2 [\mathrm{G}]^2} \tag{16}$$

An experimentally generated isotherm is obtained by plotting ΔA at a specific wavelength against the total guest concentration (ΔA *vs.* $[\mathrm{G}]_\mathrm{t}$). ΔA at each experimentally performed concentration of $[\mathrm{G}]_\mathrm{t}$ is estimated by approximately solving eq. (11) for [G] by Newton-Raphson method. A curve fitting of estimated ΔA to experimentally obtained ΔA at each point by a least square method with setting approproate initial values to K_1, K_2, $\varepsilon_{\mathrm{HG}}$ and $\varepsilon_{\mathrm{HG2}}$ can finally give K_1 and K_2.

2. Job plots of ICD spectral responses

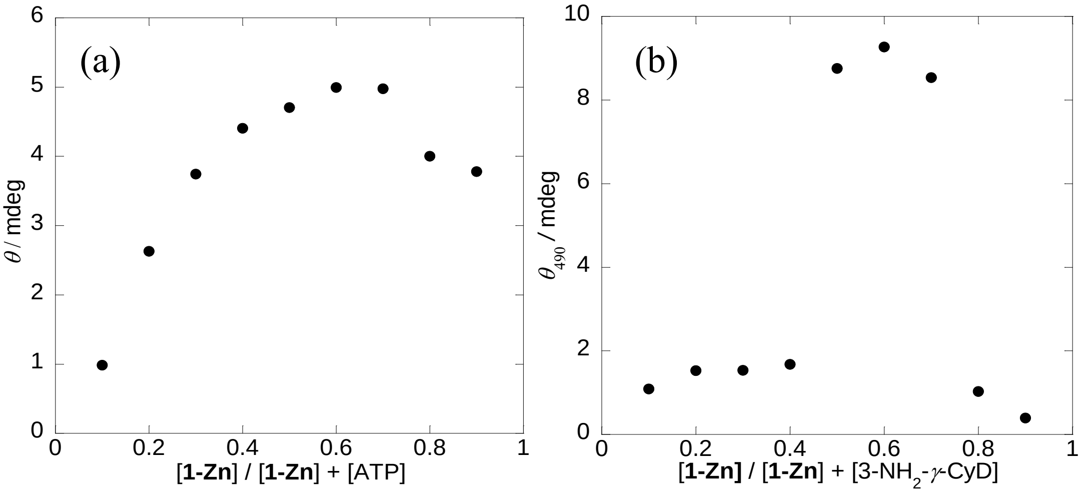

Figure S1 (a) Job plots of ICD spectral response of **1-Zn** and ATP in the presence of 3-NH_2-γ-CyD. [**1-Zn**] + [ATP] = 0.10 mM, [3-NH_2-γ-CyD] = 4.0 mM, [HEPES] = 10 mM, in 5% DMSO aq. at pH 7.4. (b) Job plots of ICD spectral response of **1-Zn** and 3-NH_2-γ-CyD in the presence of ATP. [**1-Zn**] + [3-NH_2-γ-CyD] = 0.080 mM, [HEPES] = 10 mM, [ATP] = 4.0 mM, in 4% DMSO aq. at pH 7.4.

3. H-H COSY spectrum

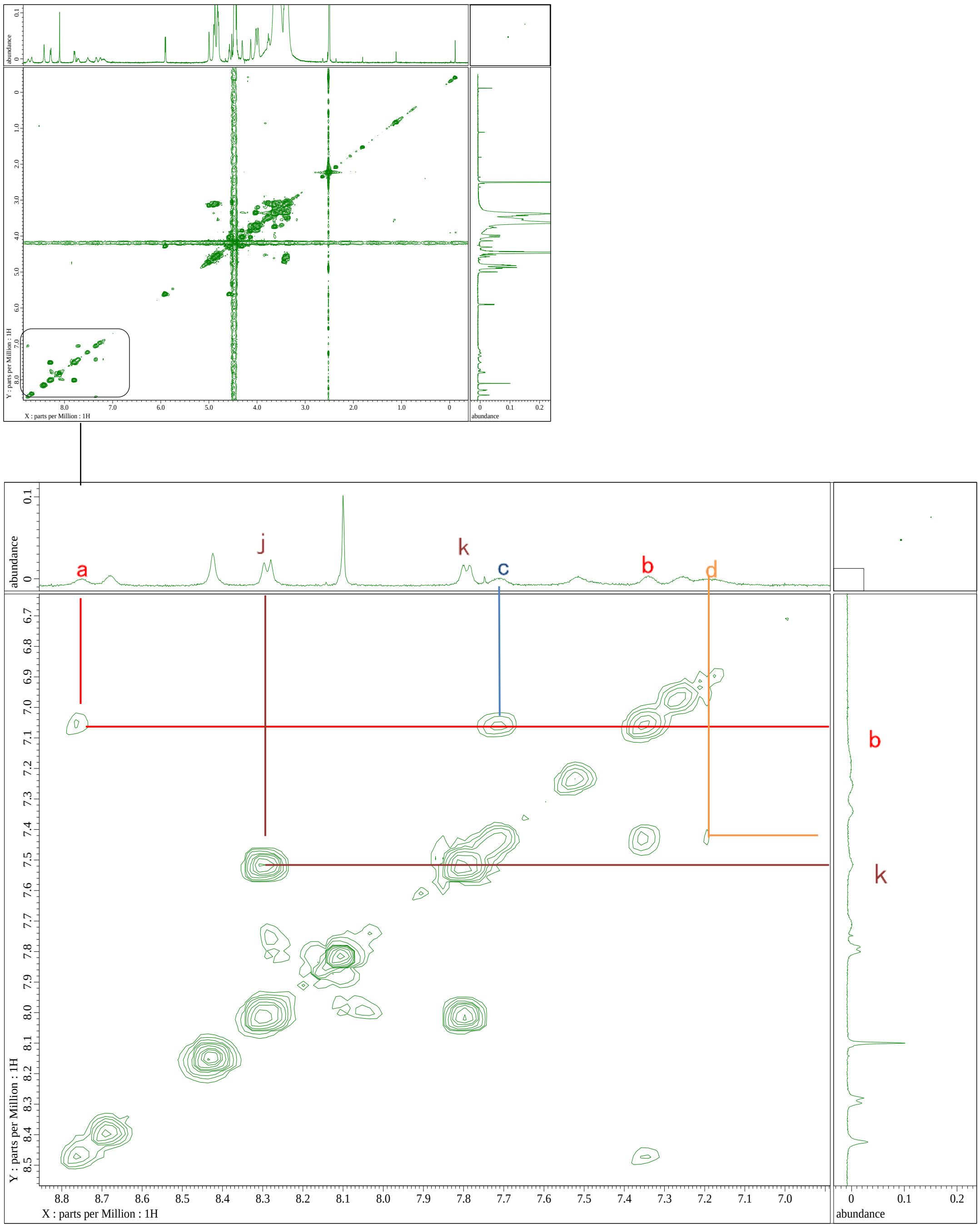

Figure S2 H-H COSY spectrum of **1-Zn**/3-NH_2-γ-CyD/ATP (22°C, 64 scans).

4. Spectral Changes of 1-Zn with addition of 3-NH$_2$-γ-CyD

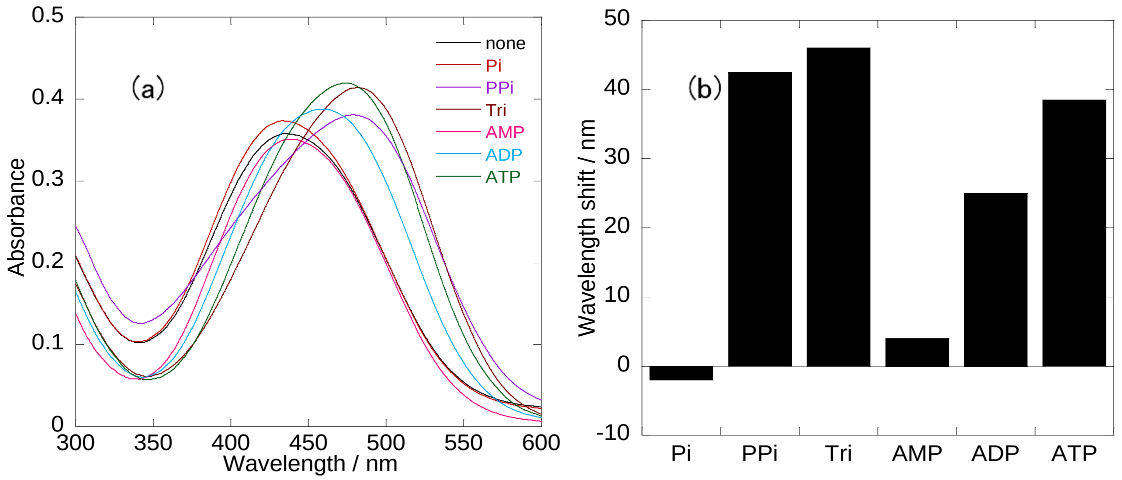

Figure S3 (a) Changes in UV-Vis spectra and (b) Wavelength shift (from 435.5 nm) of **1-Zn** upon addition of phosphate derivatives in the presence of 3-NH$_2$-γ-CyD. [**1-Zn**] = 0.020 mM, [3-NH$_2$-γ-CyD] = 2.0 mM, [phosphate] = 2.0 mM, [HEPES] = 5 mM, in 1% DMSO aq. at pH 7.4.

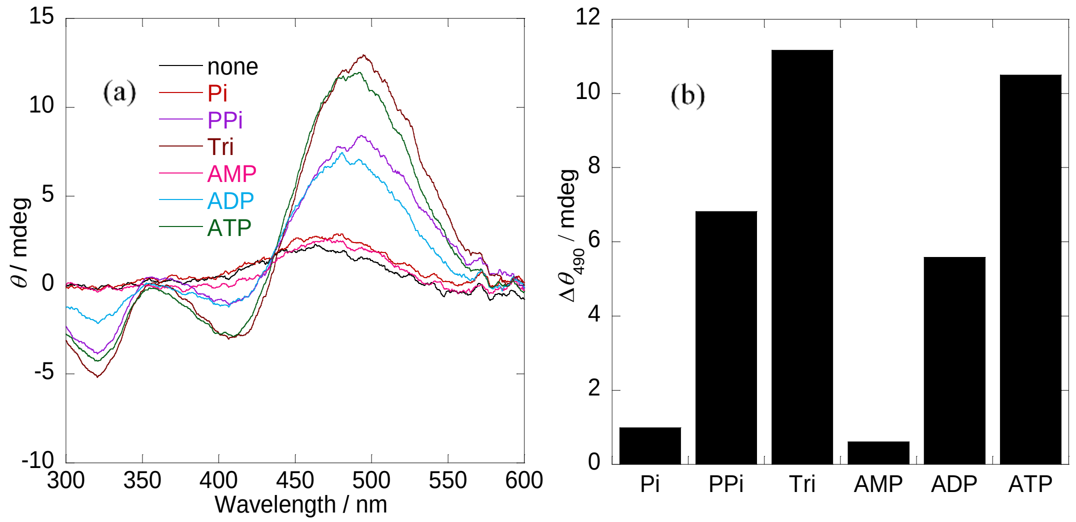

Figure S4 (a) Change in ICD spectra (b) Change of ICD intensity ($\Delta\theta_{490}$) of **1-Zn** upon addition of phosphate derivatives in the presence of 3-NH$_2$-γ-CyD. [**1-Zn**] = 0.040 mM, [HEPES] = 5 mM, [3-NH$_2$-γ-CyD] = 5.0 mM, [phosphate] = 4.0 mM, in 2% DMSO aq. at pH 7.4.

5. Identification of 1

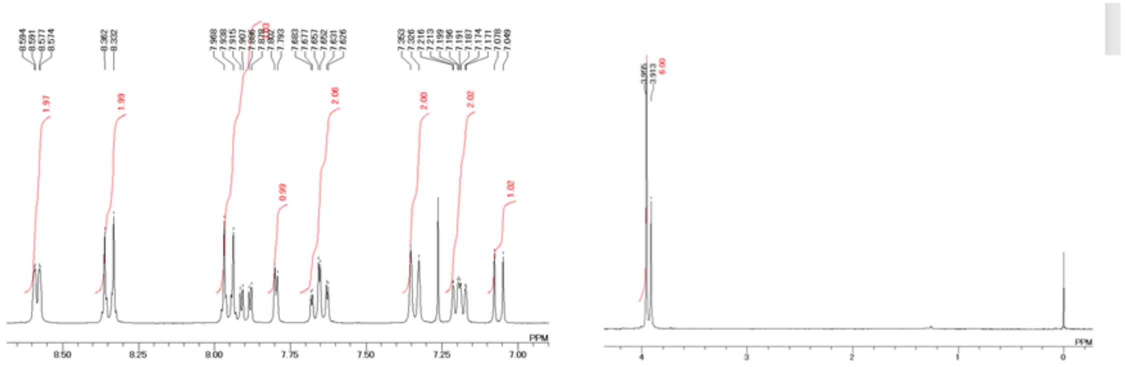

Figure S5 ^{1}H NMR spectrum of **1** (solvent: $CDCl_3$).

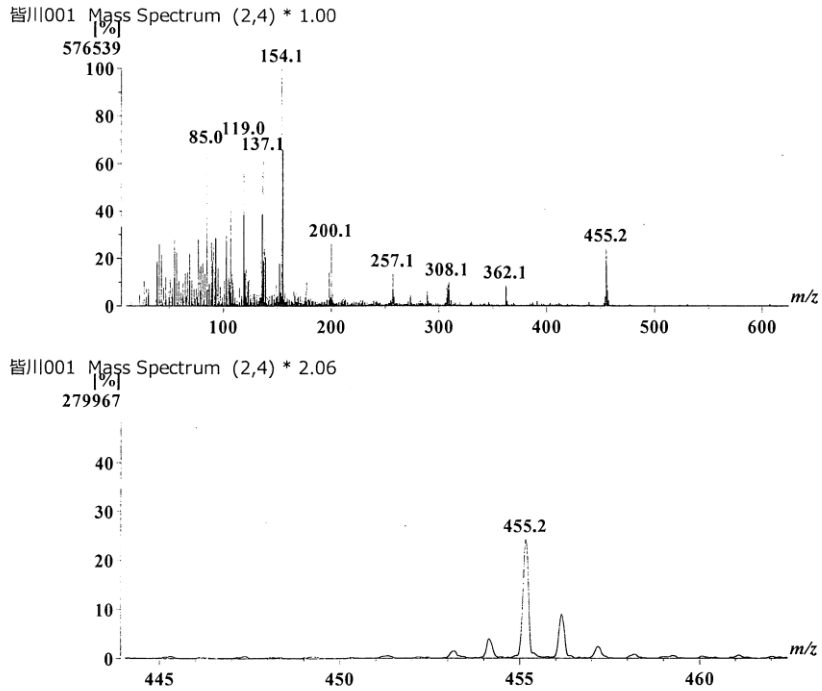

[Theoretical Ion Distribution]

Molecular Formula : C25 H22 N6 O3

(m/z 454.1753, MW 454.4881, U.S. 18.0)

Base Peak : 454.1753, Averaged MW : 454.4863(a), 454.4870(w)

```
m/z        INT.
454.1753 100.0000*************************************************************
455.1782  30.1242*****************
456.1809   4.9801***
```

Figure S6 FAB-Mass spectrum of **1**.
